# Psychological effects on patient’s relatives regarding their presence during resuscitation

**DOI:** 10.15171/jcvtr.2017.19

**Published:** 2017-06-29

**Authors:** Hassan Soleimanpour, Jafar Sadegh Tabrizi, Asghar Jafari Rouhi, Samad EJ Golzari, Ata Mahmoodpoor, Robab Mehdizadeh Esfanjani, Maryam Soleimanpour

**Affiliations:** ^1^Tabriz Health Services Management Research Center, Tabriz University of Medical Sciences, Tabriz, Iran; ^2^Students’ Research Committee, Tabriz University of Medical Sciences, Tabriz, Iran; ^3^Cardiovascular Research Center, Tabriz University of Medical Sciences, Tabriz, Iran; ^4^Neurosciences Research Center, Tabriz University of Medical Sciences, Tabriz, Iran; ^5^Social Determinants of Health Research Center, Tabriz University of Medical Sciences, Tabriz, Iran

**Keywords:** Cardiopulmonary Resuscitation, Anxiety, Depression

## Abstract

***Introduction:*** Presence of family and patients’ relatives throughout resuscitation procedure is one of the most challenging concerns.

***Methods:*** In an interventional (quasi-experimental) study that was conducted during a 6 months period, the patients’ relatives were randomly divided into two groups of intervention (the relatives who were eager to be present throughout the resuscitation procedure- under the family protection protocol, all of the procedure steps were explained to the relatives by an expert nurse who was not involved in the resuscitation procedure and control group (those who were not invited routinely to be present throughout the resuscitation procedure. However, if the control group were eager to be present, they were allowed to observe the procedure (these people were not supported by the protocol). After 90 days, subjects were contacted through telephone and filled standard questionnaires (Hospital Anxiety and Depression Scale [HADS]) and Impact of Event Scale (IES) were completed for all subjects. These questionnaires focus on anxiety, depression and post-traumatic stress disorder (PTSD). The obtained data were analyzed.

***Results:*** One hundred thirty three relatives were divided into two groups of control (59 subjects) and intervention (74 people). No significant difference was observed between two groups regarding demographic features. The evaluation after 90 days revealed depression, anxiety disorders and PTSD to be significantly more prevalent in control group than the intervention group (*P *< 0.0001 ).

***Conclusion:*** Emotional and psychological support and intervention on the patients’ relatives are efficient and can prevent the emergence of psychological disorders.

## Introduction

Presence of patient’s relatives during resuscitations has been always a matter of controversy. Relatives are rarely asked to be present in resuscitation room unless they are tended to do so.^1-6^ In emergency ward of Imam Reza hospital, Tabriz University of Medical Sciences (TUOMS) relatives are routinely permitted to be in resuscitation room if they want. However, the one who is able to serve psychological support for these people away from resuscitation procedure can be rarely found. Therefore, in present study it was tried to posit a person who can provide patients’ relatives with psychological supports in order to study the effect of such intervention on psychological conditions of relatives and then we compared them with those who were lacking the support.

## Materials and Methods

The study was in a type of interventional (quasi-experimental) study that carried out upon patients’ close relatives during a 6 month period in Imam Reza hospital, Tabriz University of Medical Sciences (TUOMS). It took 6 months to census the participants. We allocated control and intervention group in the first and second quarters of th year quarter, respectively. Inclusion criteria were: cardiac arrest cases and 18 years age or more (patient’s close relatives with 18 years or more who were patients’ wife/husband or parents or brother/sister). Exclusion criteria were: not being cooperative or having no contact with relatives or any cardiac arrest, patients without CPR (those patients who did not undergo CPR). Psychological disorders of relatives were also other exclusion criterion and were diagnosed based on patient medical history, clinical observation and consultation with a trained psychologist if it is was needed. If disorder was not identified former to resuscitation and was identified throughout or later, then the participant was excluded from the study. Studied samples were patients’ relatives who were divided into 2 groups.

### Intervention group

The group who wanted to be present during resuscitation and were informed by a professional nurse about CPR process according to a protocol. Among them, those who did not want to be present were guided to another room. Through a 2-hour workshop the nurse was taught about how to interview with relatives according to the standard instructions.

### Control group

The participants who were not routinely asked to be present during CPR. But if they were tended to be present they were permitted but were not supported by a member of CPR group during the process.

Ninety days after CPR, the participants (either the participants who witnessed the process or those who didn’t) were interviewed by one of research group members through a phone call with a questionnaire. The interviewer did not know what participant belonged to which group.

By filling an Impact of Event Scale (IES) and Hospital Anxiety and Depression Scale (HADS) based questionnaires interview with relatives was done. In fact, IES is a trusted touchstone when facing with traumatic incidents. It includes 15 items scored from 0 to 15 and their sums are classified from (no post-traumatic stress disorder [PTSD] to 75 that is severe PTSD). In such scoring system, scores 0-8 belong to subclinical area, 9-25 to mild disorder, 26-43 to average disorder and scores above 44 are considered to be severe disorders.

On the other hand, HADS questionnaire consists of two sections. One was to assess stress (7 items) and the other for depression (7 items). Scores were 0 to 21 in which 0 represented absence of disease and 21 for severe stress. Classifications in questionnaire were as follows: 0-7 no disease, 8-10 borderline and 11 for disease. Those who remained unanswered after 15 phone calls were excluded from the study. There was another questionnaire about patients’ demographic information, probable diagnosis and personal info of relatives that was filled during CPR.

After filling questionnaire data were analyzed by SPSS version 16 and demographic variants were reported in form of mean ± SD and percentage according to variants. For comparing the qualitative variables between two groups the chi-square test was used. For comparing the quantitative variables between two groups *t* test and Mann-Whitney U-test was used according to the distribution of the data. *P* < 0.05 was considered to be meaningful.

In a statistical analysis and to calculate relative risk, IES scoring was defined as mild disease (subclinical and mild) and severe disease (average and severe). On the other hand, HADS scoring was defined as absence of disease (no disease or borderline) and disease.

## Results

Participating relatives were 133. Among them 74 individuals went to intervention and 59 to control group. In intervention 45% were men and 54% were women and in control it was 66% and 34% respectively. Age average in intervention was 20-66 (40.45±10.27) and in control it was 20-63 (40.42±10.36). All participants (100%) of both groups answered yes when they were asked about whether they want to be present during CPR. Therefore, those cases who did not want to be present were excluded from the study and investigations only happened inside the two groups.

No one among relatives of both groups had a background about psychiatric disorder nor was they treated with psychiatric drugs. Those who had such criterion were excluded from the study. Demographic features of patients and relatives personal info are shown in Table 1. There was no meaningful relationship in terms of demographic features between two groups.

**Table 1:** Demographic features of patients and their relatives in control and intervention group

** Patients’ Profile**	** Intervention (n=74) **	** Control (n =59) **	**P value**
**Relative’s diseases**			
Obstructive pulmonary diseases	9	8	0.811
Heart disease	6	9	0.196
Cancer or malignancy	13	16	0.185
Renal failure	12	9	0.880
Pulmonary diseases	9	9	0.605
Diabetes	15	13	0.804
Hypertension	16	13	0.954
**The initial rhythm**			
Ventricular fibrillation	9	8
Asystole	56	42	NA
Pulseless electrical activity (PEA)	1	5
**Cause of cardiac arrest**			
Trauma	8	8
Organic disease	28	19	0.892
Predictable death	23	20
Sudden death	15	11
Patients’ profile	Intervention	Control
**Type of relative**			
Children	57	42
NA
Parents	0	4	NA
Sister/brother	12	8
Spouse	5	4
**Marital status**			
Single	10	6	NA
Married	58	52
Divorced	2	0
Widow	4	0
**Education**			
Illiterate	14	6
Diploma/ high school	41	34	0.438
Higher education	19	16
History of Former grief	23	31	0.009
Witnessing cardiac arrest	45	50	0.001

In intervention group, 85.5% of patients who had CPR died while it was 75.5% about control group. Only one patient in control group survived till 28 days after CPR. Age average of CPR patients in intervention was 65.67±14.7 and in control it was 63.15±16.32 which was not meaningful. Relatives’ mental condition was studied 90 days after CPR by IES and HADS questionnaires.

In intervention group there was not a meaningful linkage between relatives age and PTSD severity (*P =* 0.705 and r= 0.045). Similarly, no meaningful difference was found in control group (*P =* 0.739 and r=0.044).

Moreover, in both groups, the meaningful relationship was not established between relatives’ age and anxiety/depression severity (*P* = 0.137 & r=0.174 and *P* = 0.341 & r=0.126 for anxiety and *P* = 0.174 & r=0.160 for depression respectively).

No meaningful relation also was observed between severity of anxiety/depression and PTSD and relatives’ gender in both groups.

IES questionnaire, dealing with study of PTSD among relatives, showed that in control PTSD was meaningfully more than intervention (*P* < 0001) (Table 1).

Compromised in two sections, HADS questionnaire was allocated to depression in its first part and the second part concentrated on relatives’ anxiety. It revealed that after 90 days of CPR depression in control group was meaningfully higher than intervention (*P* < 0001) (Table 2) the same axiom was also found when anxiety issue came through (*P* < 0.0001).

**Table 2:** The comparison of anxiety, depression and PTSD in 25-50 and 75 quartets

		**Intervention**	**Control**	***P* value**
	First quartile	4	10	
Anxiety	Median	6	13	<00001
	Third quartile	8	18	
	First quartile	3/75	8	
Depression	Median	6	10	<00001
	Third quartile	28.8	11	
	First quartile	5	32	
PTSD	Median	10	46	<00001
	Third quartile	15	49	

Table 2 shows that depression; anxiety and PTSD in 25, 50 (medium) and 75 quartiles are meaningfully higher in control group compared to the intervention.

Considering disorder severity the relative risk of severe PTSD in intervention group compared to control was 0.05. CI (0.016-0.152)

The relative risk of depression in control group was also higher than intervention. RR=0.266 and CI (0.121-0.582).

In addition, control group depicted higher presence of anxiety disease than the intervention. RR=0.074 and CI (0.028-0.195).

## Discussion

Presence of relatives during CPR has been scrutinized globally in different studies. The results came to be various as cultures and attitudes vary. But the common language of literature suggested a high tendency among relatives to be present during CPR. They also express that psychological and mental supports have direct and obvious impact on relatives’ future life of who perceived CPR process.

Results of our study also suggested the desire among relatives to be present during CPR. It also confirmed the impact of supports and intervention on prevention of depression, anxiety and PTSD.

Presence of relatives during CPR has been divided in to 3 categories:

1) Being present
2) Satisfaction
3) Coping with presence consequences

### Being present

In an investigation by Meyers et al, 80% relatives wanted to be present during CPR.^7^ In other several studies^3^ the authors indicated that patients’ relatives wanted to be present again if other future CPRs would happen. In these researches, relatives declared that being present is their essential right and it can be effective either for themselves or to the patient. In a study by Holzhauser et al, they showed that even 72% of control group members believed if they were present during CPR they would be able to better cope with their stress.^8^ In another research in Pakistan on 290 relatives whose patients were died after CPR^8^ 98% said that patients’ relative should be permitted to be inside CPR room. However, only 52% believed that if they were in CPR room they would be helpful.^9^ In opposition to other studies, our investigation revealed that all relatives tended to be present during CPR.

### Coping

Meyers et al came to the conclusion in their study that the presence of relatives in CPR made them to forget memories about the accident even after 2 months. In another investigation by Robinson et al^10^ they showed that psychological and mental supports of the relatives didn’t make any difference in the emergence of psychological disorders. In present study due to small sample size we didn’t run an accurate statistical analysis with an appropriate sample power. In a study by Lesk and Brasel^11^ on two groups who witnessed CPR (family witnessed resuscitation, FWR) and didn’t witnessed CPR, they suggested that the level of symptoms such as coping, feeling wellbeing and problem solving were similar in both groups that connote no meaningful differences. However, the study took place among traumatic patients (accident or bullet wounded cases) whose relatives, either they were present during CPR or not, suffered more than chronic relatives. Another issue about the study was that interviewing with relatives took place only two days after CPR which made it hard to estimate the impact of relatives’ presence during CPR. Another disadvantage was the small sample size of their study.

A research by Campton et al^12^ on two groups of relatives (FWR and Non-FWR) conferred that PTSD level after one month of accident was two times higher in witnessing group than non-witnessing. However, no kind of supportive intervention was carried out upon witnessed relatives.

In another study by the same researcher,^13^ similar findings were obtained. He divided 65 participants into two groups of FWR and non-FWR and studied them in 30 and 60 days after CPR in terms of PTSD and depression and found no meaningful difference among them. Again there was no kind of supportive intervention in FWR group in their study. While in our study there was a group whose members were completely supported by a trained person outside CPR process.

In agreement with our study Jabre et al^14^ who investigated 570 relatives showed that PTSD level and anxiety disorder was less in a group who were under psychological and mental support during CPR. They suggested that PTSD level, anxiety and depression in CPR witnessed group after 90 days of CPR was meaningfully less than non-witnessed group. In our study those participants who wanted not to be present during CPR were excluded and it made impossible for us to study this issue. As a continuum to the study of Jabre et al, another research was carried out one year later upon the same groups. This time, 408 relatives were investigated about PTSD, anxiety and depression and also grief disorder. Structure of groups in terms of intervention and control was similar. Even after one year, those who gained psychological and mental supports had meaningfully lower PTSD, anxiety and grief disorder levels. The study also suggested that relatives who were lucky to be present during resuscitation had meaningfully less disorders compared to those who were absent.^15^

However, there are controversies about individuals’ adaptation after several days or months in various studies. Those studies that were conducted shortly after patient’s death did not show the impact of relatives’ presence during CPR. Unlikely, studies with acceptable intervals after grief suggested positive impact of relative’s presence. But the controversies may go back to the differences in the way the plan is administered or presence of mental supports and their quality during CPR. But it is clearly demonstrated that by great intervals in investigations we will have more positive effects of psychological and mental supports on relatives’ anxiety, depression and PTSD level. Hence the role of medical staffs is undeniable.

The most important limitation of our study was the relatively small sample size. On the other hand our study was conducted in only one center. Thus, the results derived from this study cannot be generalized to all populations.

## Conclusion

Relatives’ presence during CPR and providing them with psychological and mental supports by experts and trained staffs can play vital role in reduction of their stress and psychological disorders after accidents. In conclusion, it is necessary to initiate giving them psychological supports to reduce their disorders.

## Competing interests

The authors declare no conflict of interest.

## Ethical approval

The study was confirmed by committee of ethics of TUOMS (No: 9367). Written informed consent was achieved from all the relatives participating in emergency department.

## Acknowledgments

The authors would like to express their gratitude towards participating nurses, Nahid Taghizadegan, Aida Nazari, Zahra Hasanlou, Khadijeh Asghari, Parinaz Zirak, Monireh Beheshtizade and Farideh Hooshmand from Tabriz Medical University for their assistance to interview with patients’ relatives. This article is based on a dataset forming part of Asghar Jafari Rouhi’s specialty thesis, entitled “Psychological Effects on Patient’s Relatives regarding their Presence during Resuscitation”. It is registered at Tabriz University of Medical Sciences (No: 93.3–6.8) and was presented in June 2015. This article was supported by Tabriz Health Services Management Research Center, TUOMS, Tabriz-51664, I.R., Iran. Special thanks to Research Vice Chancellor of TUOMS for all the material and financial support in our study.
